# Comment on Deeb *et al.*: interpretation of breath cannabinoids and implications for impairment

**DOI:** 10.1093/jat/bkag028

**Published:** 2026-05-06

**Authors:** Aaron Olson

**Affiliations:** ARO Consulting LLC, Hugo, MN 55038, United States

## Abstract

This letter addresses the article by Deeb *et al.* describing the analysis of cannabinoids in exhaled breath. While the study represents a technical advancement in breath-based detection, several interpretive and analytical limitations warrant clarification. The manuscript discusses potential impairment-related applications despite the absence of direct measures of impairment. Existing literature, including controlled clinical studies and government reports, indicates that THC concentrations in biological matrices cannot be used as a reliable indicator of impairment. Additionally, key variables influencing cannabinoid exposure, including dose, potency, and inhalation behavior, were not controlled, further limiting interpretability. Analytical concerns are also noted, including subpar chromatographic resolution (Rs ≈ 0.9), which may compromise accurate identification and quantitation of structurally similar cannabinoids. These issues highlight the need for caution in interpreting breath cannabinoid data, particularly in forensic contexts where conclusions regarding impairment may carry significant consequences.

Dear Editor,

I commend the authors for their work in their latest paper entitled “Simultaneous Analysis of Δ9-THC, Δ8-THC, CBD, and CBN in Breath Aerosols Collected Using Cannabix Technologies Breath Collection Unit [1].”

While the study represents a meaningful technical effort, several aspects of the interpretation, analytical approach, and experimental design warrant further comment.

A primary concern relates to the manuscript’s framing of breath cannabinoid detection in the context of impairment. The study does not include any direct measurement of impairment, performance, or cognitive function. No driving simulation, behavioral testing, or validated impairment metrics are presented. Despite this, the authors imply relevance to impairment-related determinations. However, the cited literature does not establish such a relationship [2–4]. For example, Lynch *et al.* explicitly note that “connection to level of impairment has not yet been investigated,” despite demonstrating detectability and correlation with blood concentrations [3].

In contrast, a substantial body of research directly addresses this issue and reaches the opposite conclusion. In the largest randomized, double-blinded, placebo-controlled trial to date, Fitzgerald *et al.* reported that “THC concentrations (and/or metabolites/related cannabinoids) in blood, oral fluid, or breath cannot be used as a sole indicator of impairment,” and further that “there was no correlation between THC… in blood, oral fluid, or breath and driving performance” [5]. Similarly, Compton, in his report to Congress, concluded that there is no chemical test for marijuana impairment comparable to alcohol and that THC concentrations alone do not correlate well with impairment [6].

The relationship between THC concentration and impairment is highly variable and dependent on multiple factors, including dose, potency (percent THC), route of administration, inhalation timing, and user tolerance [7]. In the present study, cannabis was self-administered without standardization of potency or dose. The percent THC of the material was not characterized, and critical variables such as the number of inhalations, duration of breath-hold, and total dose delivered were not controlled. Each of these factors affects the exposure and pharmacodynamic response. As a result, the presence or concentration of THC in breath, even if accurately measured, cannot be used to determine impairment.

There are also important analytical concerns. The reported chromatographic resolution (Rs ≈ 0.9) reflects incomplete separation of analytes. Baseline resolution is generally understood to require Rs ≥ 1.5 to ensure accurate peak integration and reliable quantitation [8, 9]. This issue is particularly important for cannabinoids, where structurally similar isomers (e.g. Δ8-THC and Δ9-THC) must be reliably distinguished. Separation of compounds is not merely a technical preference but a necessity, particularly in light of recent forensic toxicology errors involving THC isomer misidentification and interference [10, 11].

In summary, I commend the authors for their contribution to the growing body of research on breath-based cannabinoid detection. However, the interpretation of the findings, particularly regarding impairment, should be analyzed with caution. Clarifying these issues would strengthen the manuscript and better align it with current scientific understanding and forensic practice.

Thank you for your consideration.
